# Temporal organization of gonadal and adrenal steroid fluctuations in male mice

**DOI:** 10.1530/EC-26-0091

**Published:** 2026-03-23

**Authors:** M Chase Kettering, Marion C Hope, Christian A Unger, Cassidy E Socia, Zannatul Mauya, William E Cotham, Staci Downey, Louise Lantier, Reilly T Enos

**Affiliations:** ^1^ Department of Pathology, Microbiology, and Immunology, University of South Carolina-School of Medicine, Columbia, South Carolina, USA; ^2^ Department of Chemistry and Biochemistry, College of Arts and Science, University of South Carolina, Columbia, South Carolina, USA; ^3^ Vanderbilt Mouse Metabolic Phenotyping Center, Department of Molecular Physiology & Biophysics, Vanderbilt University, Nashville, Tennessee, USA

**Keywords:** testosterone variability, corticosterone, progesterone, deoxycorticosterone, adrenal glands

## Abstract

**Graphical Abstract:**

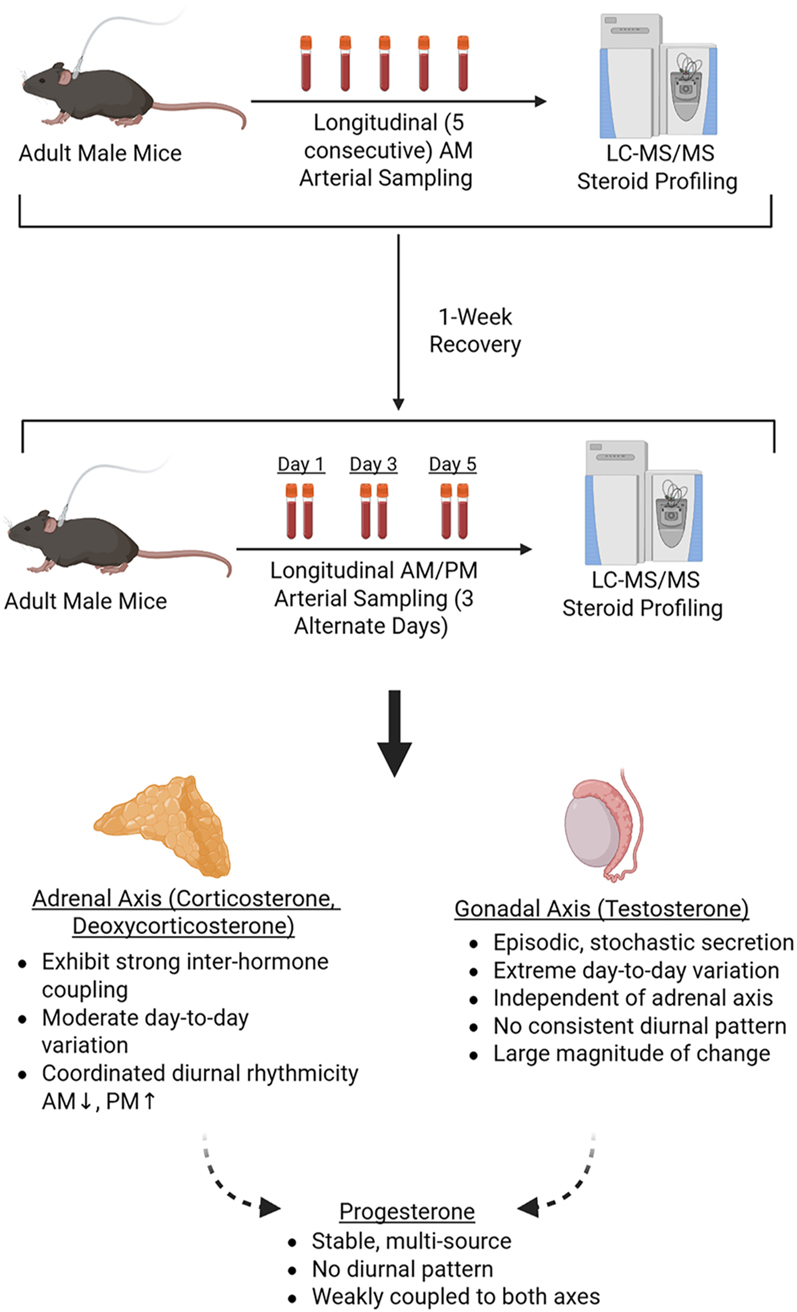

Longitudinal arterial sampling combined with LC–MS/MS profiling was used to define the temporal organization of circulating steroid hormones in adult male mice. Adrenal steroids (corticosterone and deoxycorticosterone) exhibited coordinated diurnal rhythmicity, whereas testosterone secretion was highly variable and episodic, showing no consistent diurnal pattern. In contrast, progesterone remained comparatively stable and was weakly coupled to both adrenal and gonadal steroid dynamics. Created with Biorender.

**Abstract:**

Steroid hormones exhibit temporal fluctuations that influence physiology, yet these dynamics remain incompletely characterized in male mice, a cornerstone model in endocrine research. Using serial arterial sampling and high-sensitivity liquid chromatography–tandem mass spectrometry (LC–MS/MS), we performed the first longitudinal, multi-analyte quantification of steroid hormones (testosterone, progesterone, corticosterone, and deoxycorticosterone (DOC)), within the same mice across consecutive days and diurnal periods. Adult male C57BL/6J mice (*n* = 13) were sampled daily for 5 days and again at 7 AM and 5:30 PM on non-consecutive days (days 1, 3, and 5). Corticosterone and its adrenal precursor – DOC – exhibited coordinated diurnal patterns, with corticosterone showing consistent AM–PM increases across all sampling days and DOC reaching significance only on day 5. These findings confirm a circadian pattern of adrenal steroidogenesis dominated by corticosterone, with its faster-turnover intermediate displaying less stable rhythmicity. In contrast, testosterone fluctuated irregularly and without a consistent AM–PM pattern, varying up to 100-fold within individual mice. Progesterone showed minimal day-to-day or diurnal variation. Correlation analyses revealed tight coupling among corticosterone and DOC (*r* = 0.99, *P* < 0.001), but no association between adrenal steroids and testosterone, indicating independent regulation of adrenal and gonadal axes. These results define the natural temporal organization of steroid hormones in male mice, distinguishing coordinated adrenal rhythmicity from pulsatile gonadal variability, and provide a physiologic framework for experimental design and data interpretation in murine endocrine research.

## Introduction

Steroid hormones regulate metabolism, reproduction, stress responses, and circadian physiology (1, 2, 3, 4, 5, 6). Although circulating steroid levels are well characterized in humans (7, 8, 9), less is known about their natural temporal variability in male mice, despite the species’ widespread use in mechanistic and preclinical studies (1, 2, 3, 4, 5, 6).

Earlier rodent studies described pulsatile testosterone secretion in male mice and demonstrated that testosterone concentrations can vary markedly both between animals and within the same animal sampled on different occasions (10, 11, 12, 13). However, these studies relied on immunoassay-based detection, single-hormone measurements, and either cross-sectional sampling or short-duration time series. As a result, they could not resolve how gonadal hormone variability relates to other steroidogenic pathways within the same mouse.

Liquid chromatography–tandem mass spectrometry (LC–MS/MS) now provides the gold-standard method for steroid analysis, offering unmatched specificity and quantitative precision while eliminating antibody-based cross-reactivity. Importantly, prior evaluations have demonstrated that commonly used testosterone immunoassays exhibit a substantial method-specific bias and limited accuracy when applied to mouse serum and steroidogenic tissue extracts, whereas LC–MS/MS provides the most reliable quantification across the full physiological range of testosterone concentrations in mice (14).

These advances enable the reliable detection of multiple low-abundance steroids from small plasma volumes, allowing comprehensive endocrine profiling within the same mice (15, 16, 17, 18, 19, 20, 21, 22). Building on this progress, sex steroid fluctuations were recently characterized not only in serum but also across 15 different tissues throughout the murine estrous cycle, as well as in ovariectomized mice, highlighting temporal variation in hormone levels (20).

Here, we used serial arterial sampling combined with LC–MS/MS to perform the first longitudinal, multi-analyte assessment of steroid hormones within the same individual male mice across consecutive days and diurnal timepoints. The objective of this approach was to define the temporal organization and coordination of adrenal and gonadal steroid hormone dynamics under physiologically stable conditions. We quantified testosterone, progesterone, corticosterone, and its adrenal intermediate – deoxycorticosterone (DOC) – to capture both gonadal and adrenal components of steroidogenesis. This comprehensive approach enables direct comparison of intra-mouse variability, diurnal rhythmicity, and inter-hormone relationships under physiologically stable conditions. The resulting dataset defines, for the first time, the structure, magnitude, and correlation of circulating steroid fluctuations in adult male mice and establishes a physiological reference for experimental endocrine studies.

## Methods

### Experimental design

12-week-old male C57BL/6J mice (*Mus musculus*) (*n* = 13) were purchased from Jackson Laboratories (USA) and housed in a temperature- and light-controlled facility (21 ± 1°C, lights on from 6 AM–6 PM). Mice were fed the purified, open-source AIN-76A diet. After one week of acclimation to the facility, mice underwent carotid artery catheterization under isoflurane anesthesia. Following surgery, mice were singly housed and allowed a one-week recovery period. Blood samples were then collected daily over a consecutive five-day period at 7 AM. After another week of recovery, additional blood samples were collected at both 7 AM (1 h after lights on) and 5:30 PM (30 min prior to lights off) on non-consecutive days, totaling three collection days. Blood sampling was performed using an indwelling carotid artery catheter connected to a subcutaneously implanted vascular access button (Instech Laboratories, USA) positioned in the intrascapular region. Mice were connected to the sampling system only at the time of blood collection. For each sampling event, the access port was briefly connected to a sterile catheter (∼15 cm in length) attached to a sampling syringe. Mice were not scruffed, restrained, or removed from their home cage at any time. The cage was opened, and the mouse was briefly touched only to connect the catheter to the access port, a process that required only a few seconds. Once connected, blood was withdrawn from outside the cage while the mouse freely moved within its home cage, minimizing handling-associated stress. Blood collection required approximately 30–60 s, yielding ∼25–30 μL of plasma per sampling event. Following collection, the catheter was flushed with sterile heparinized saline (10 mU/mL; ∼20–40 μL) to maintain patency and then disconnected. The total duration of catheter connection for each sampling event was approximately 1 min. For each blood collection, mice were connected to the sampling system.

### Mass spectrometry

Sample processing and LC-MS/MS were performed as previously described in detail using a Q Exactive HF-X Hybrid Quadrupole-Orbitrap mass spectrometer with a Vanquish HPLC on the front end (Thermo Electron, USA) (20). We probed for testosterone, progesterone, corticosterone, DOC, androstenedione, 17*β*-estradiol (E2), and dihydrotestosterone (DHT). The lower limit of quantification (LLOQ) for each analyte was established during method validation using 200 μL extraction aliquots of charcoal-stripped fetal bovine serum and is therefore reported as absolute mass per 200 μL to reflect the validated assay conditions. For clarity and comparison with published literature, the equivalent concentration values (pg/mL) are also provided by normalizing the 200 μL mass to 1 mL (i.e. multiplying by 5): testosterone 0.5 pg per 200 μL (2.5 pg/mL), progesterone 0.5 pg per 200 μL (2.5 pg/mL), deoxycorticosterone (DOC) 0.25 pg per 200 μL (1.25 pg/mL), 17*β*-estradiol 0.5 pg per 200 μL (2.5 pg/mL), and dihydrotestosterone 10 pg per 200 μL (50 pg/mL). These concentration equivalents assume linear scaling and equivalent extraction efficiency and are provided for interpretive purposes. However, as expected, due to limited sample volume, only testosterone, progesterone, corticosterone, and DOC were reliably detected and were included in the results of this investigation. Prior to processing samples, internal standards (Sigma-Aldrich, USA) for testosterone (−2,3,4-^13^C_3_), progesterone (D_9_), corticosterone (−^13^C_3_) (also used for DOC), E2 (D_5_), androstenedione (−2,3,4-^13^C_3_), and DHT (16,16,17-D_3_) were added to each serum sample before plasma processing to correct for extraction efficiency, matrix effects, and ion suppression in each individual sample. Calibration curves utilizing a certified reference material (testosterone, Sigma-Aldrich, Catalog No. T037; progesterone, Sigma-Aldrich, Catalog No. P-069; corticosterone, Sigma-Aldrich, Catalog No. C-117; DOC, Sigma-Aldrich, Catalog No. D-105) were used to determine the quantity of each steroid. Representative chromatograms presented for testosterone, progesterone, corticosterone, and deoxycorticosterone (DOC), along with corresponding internal standards, are presented in Supplementary Figs 1 and 2 (see the section on Supplementary materials given at the end of the article), respectively, with signal intensities normalized to 100%. Although formal assay validation was performed using 200 μL extraction volumes, the high analytical sensitivity of the method permitted reliable quantification from the ∼25–30 μL plasma volumes obtained during serial arterial sampling. All samples were analyzed within a single analytical run to eliminate inter-assay variability. The run included three method blanks and low- and high-concentration quality control samples for each analyte to assess intra-assay variability. Intra-assay coefficients of variation were <3% for all analytes measured. Because of the wide biological variability observed among mouse samples, particularly for testosterone, calibration was performed using weighted standard curves (1/*x*) to ensure accurate quantification across the full physiological concentration range. All calibration curves demonstrated excellent linearity (coefficients of determination, *R*^2^ ≥ 0.98). Testosterone, progesterone, corticosterone, DOC, and their stable isotope labeled internal standard were analyzed using parallel reaction monitoring mode fragmenting the M + H ion in the HCD collision cell and measuring the fragments in the Orbitrap analyzer. For testosterone, M + H precursor ion = 289.2162 with quantifying product ion 97 *m*/*z*; for ^13^C_3_ testosterone, M + H precursor ion = 292.2263 with quantifying product ion 100 *m*/*z*; for progesterone, M + H precursor ion = 315.2319 with quantifying product ion 97 *m*/*z*; for D_9_ progesterone, M + H precursor ion = 324.2884 with quantifying product ion 100 *m*/*z*; for corticosterone, M + H precursor ion = 347.2217 with quantifying precursor ion 121 *m*/*z*; for DOC, M + H precursor ion = 331.2268 with quantifying precursor ion 97 *m*/*z*; and for ^13^C_3_ corticosterone, M + H precursor ion = 351.2468 with quantifying product ion 121 *m*/*z*.

### Statistical analysis

Data were analyzed using Prism 10 statistical software (GraphPad Software, USA). For the five-day daily steroid evaluation, changes in hormone levels over time were assessed using a one-way repeated measures ANOVA, followed by the Newman–Keuls post hoc test. To evaluate differences in the coefficient of variation (CV) for each analyte within each animal during the five-day period – as well as to assess the mean magnitude of change observed in the AM/PM sampling experiment – a one-way ANOVA with the Newman–Keuls post hoc test was also performed. To evaluate within-animal variability during the AM/PM sampling experiment, we quantified the non-directional magnitude of diurnal change for each hormone. For each mouse and for each of the three days on which both AM and PM samples were collected, the absolute fold difference between AM and PM concentrations was calculated irrespective of the direction (i.e. AM > PM or PM > AM). These values were then averaged within each mouse to generate a single magnitude-of-change metric per animal. This approach quantifies the amplitude of diurnal variation without assuming consistent directional differences between time points. Diurnal differences within individual days were analyzed using two-tailed Student’s *t*-tests. A Pearson correlation coefficient was used for any correlation assessment. The normality of continuous variables was assessed using the Shapiro–Wilk test. If a variable failed the normality test, the values were log10-transformed prior to statistical analysis. All statistical tests (one-way repeated measures ANOVA, two-tailed paired *t*-tests, one-way ANOVA, and Pearson correlations) were performed on the transformed data when transformation was required; otherwise, tests were performed on the raw data. For clarity, figures display hormone concentrations on a log10 scale where indicated. All data are presented as mean ± standard error (SE), and the statistical significance was defined as *P* < 0.05.

## Results

### Testosterone exhibits the highest intra-individual variability across five days

**Figure 1:**
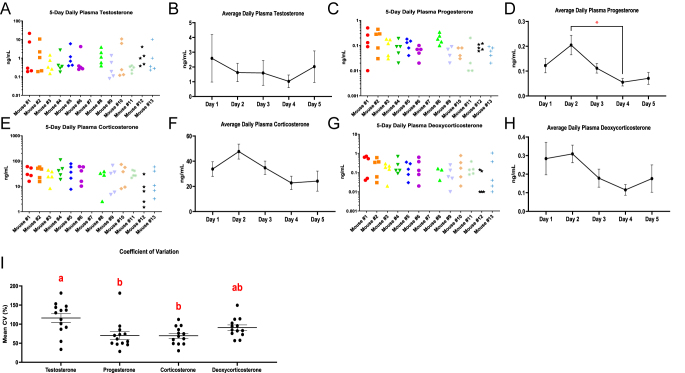
Daily fluctuations in circulating steroid hormone levels in male mice. Individual plasma concentrations over five consecutive days are shown for testosterone (A), progesterone (C), corticosterone (E), and deoxycorticosterone (DOC) (G) (*n* = 13). In these individual panels, the *x*-axis represents individual mice (mouse ID 1–13), and each mouse has five data points corresponding to days 1–5. Hormone concentrations are displayed on a log10 scale. Group mean ± SEM values across days are shown for testosterone (B), progesterone (D), corticosterone (F), and DOC (H) on a linear scale. One-way repeated measures ANOVA revealed a significant difference in progesterone between day 2 and day 4 (*P* < 0.05). (I) Coefficient of variation (CV) across 5 days for each hormone within individual animals. Groups not sharing a common letter are statistically different (*P* < 0.05).

Daily plasma concentrations of testosterone, corticosterone, and progesterone were measured in 13 adult male mice over a five-day period (Fig. 1A, B, C, D, E, F, G, H). Testosterone demonstrated the most pronounced intra-mouse variability, with several mice exhibiting fluctuations spanning over 10- to 100-fold changes between days. Despite these large changes within individual animals, no consistent group-level differences were detected across the five days, reflecting the high degree of natural biological variation both within and among animals.

Corticosterone levels showed higher absolute concentrations but more moderate within-animal fluctuations, with typical fold changes ranging between two- to six-fold. The corticosterone precursor DOC exhibited intermediate variability, generally fluctuating between two- and eight-fold across days. Progesterone was the most stable steroid measured, with fold changes generally below five-fold within individual mice across days.

Quantitative analysis of within-animal coefficient of variation (CV) values confirmed that testosterone exhibited significantly greater variability than progesterone and corticosterone (*P* < 0.01) but was not statistically different from DOC (Fig. 1I). Moreover, the CV of DOC was not significantly different from that of corticosterone or progesterone, reflecting intermediate variability among these steroids. Although progesterone concentrations showed a modest but significant difference between day 2 and day 4 (*P* < 0.05), none of the analytes demonstrated consistent group-level day-to-day trends. Collectively, these data indicate that testosterone secretion in male mice is highly episodic and stochastic, whereas adrenal steroids and progesterone exhibit more stable and moderately variable daily patterns.

### Correlations reveal coordinated adrenal steroid regulation and independent testosterone variability

**Figure 2:**
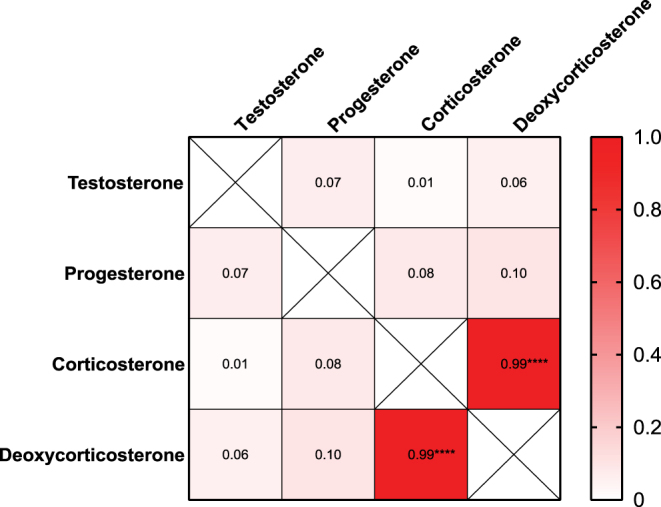
Correlations among circulating steroid hormones. Pearson correlation matrix generated from all plasma samples showing relationships among testosterone, progesterone, corticosterone, and deoxycorticosterone (DOC). Color intensity corresponds to correlation strength (scale 0–1), with darker shading indicating stronger positive correlations. Diagonal cells represent self-correlations and are marked with an “*X*.” Asterisks denote significance levels (****P < 0.0001).

To assess inter-hormone relationships and coordination among steroidogenic pathways, Pearson correlation analyses were performed using all plasma samples across timepoints (Fig. 2). As expected, strong positive correlations were observed among corticosterone and DOC (*r* = 0.99–1.00, *P* < 0.001), indicating tight coupling within the adrenal steroidogenic pathway.

In contrast, testosterone and progesterone showed no significant correlations with any adrenal-derived steroids (*r* < 0.1, *P* > 0.1), underscoring their independent regulation and lack of temporal association with adrenal rhythmicity. The absence of correlation between testosterone and corticosterone further confirms that gonadal and adrenal axes operate independently under basal physiological conditions. Together, these results demonstrate that the adrenal steroid output is highly coordinated, whereas testosterone secretion remains stochastic and temporally uncoupled from adrenal rhythms.

### Diurnal hormone profiling identifies corticosterone rhythmicity and testosterone variability

**Figure 3:**
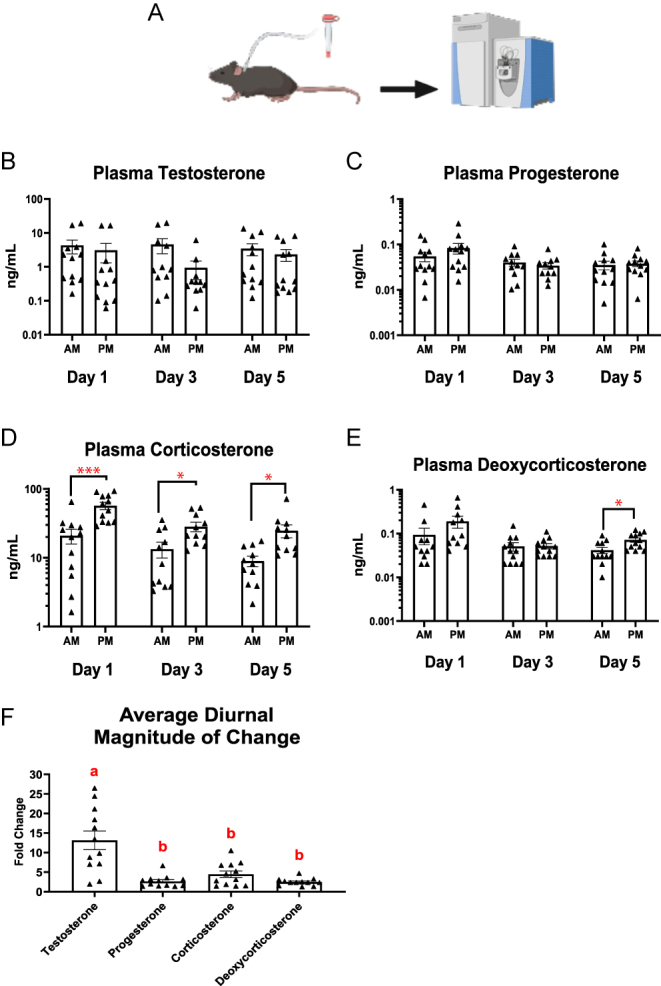
Diurnal variation in steroid hormone levels and magnitude of change across time points in male mice. (A) Experimental timeline for diurnal hormone sampling. Following the 5-day daily sampling period and 1 week of recovery, mice underwent three additional non-consecutive blood collections at 7:00 AM (1 h after lights on) and 5:30 PM (30 min prior to lights off) on days 1, 3, and 5 (*n* = 12). Paired AM and PM plasma concentrations of testosterone (B), progesterone (C), corticosterone (D), and deoxycorticosterone (DOC) (E) are shown. Hormone concentrations in panels (B, C, D, and E) are displayed on a log10 scale. (F) Average non-directional magnitude of AM–PM change for each hormone across animals and time points. Data are presented as mean ± SEM. Groups not sharing a common letter are statistically different (*P* < 0.05).

In a separate experiment, we examined diurnal variation in circulating steroid hormones by collecting blood samples in the morning (AM) and evening (PM) on days 1, 3, and 5 (Fig. 3A).

Corticosterone levels (Fig. 3D) showed a robust and statistically significant increase from AM to PM on all three days (day 1 *P* < 0.001; day 3 *P* < 0.05; and day 5 *P* < 0.05), with average PM values ranging from 2.3- to 2.9-fold higher than AM values. The adrenal precursor, DOC, also tended to rise in the evening (Fig. 3E). Although DOC exhibited higher PM than AM concentrations on all three days, statistically significant AM–PM differences were detected only on day 5 (*P* < 0.05).

In contrast, testosterone (Fig. 3B) and progesterone (Fig. 3C) did not exhibit statistically significant AM–PM differences on any day. However, testosterone demonstrated the greatest within-animal variability, with some mice showing fold changes exceeding 20-fold between morning and evening. Across all animals, the average magnitude of diurnal change (i.e., absolute fold change regardless of direction) was the highest for testosterone (13.6 ± 2.4) (Fig. 3F; *P* < 0.001).

These data reveal distinct hormone-specific temporal profiles: corticosterone and its precursor show coordinated diurnal rhythmicity, testosterone exhibits pronounced stochastic variability without directional consistency, and progesterone remains comparatively stable across the light–dark cycle. Together, these findings underscore that sampling time and expected intrinsic variability are critical considerations in the design and interpretation of endocrine studies in male mice.

## Discussion

Our research group routinely assays sex steroids in rodent samples for our own projects and for collaborators at other institutions. Notably, with testosterone data, investigators are often surprised by the high variability of testosterone fluctuations in male mice. Classic studies established that testosterone secretion in male mice is highly variable and episodic, often changing unpredictably over short time intervals (10, 11). Notably, early work also demonstrated that testosterone concentrations measured twice in the same mouse vary as much as concentrations measured across different animals sampled once, supporting the conclusion that testosterone secretion in mice is intrinsically episodic rather than reflecting stable individual set points (12). However, these studies were limited by immunoassay-based detection, single-hormone measurements, and either cross-sectional sampling or short-duration time series. As a result, they could not resolve how gonadal hormone variability relates to other steroidogenic pathways within the same mouse.

Steroid hormone secretion is regulated by distinct endocrine control systems that impose different temporal patterns on circulating hormone levels. In male mice, testicular testosterone production is driven by episodic activation of the hypothalamic–pituitary–gonadal axis, resulting in irregular, pulsatile hormone release (10, 11). In contrast, adrenal steroid production is regulated by the hypothalamic–pituitary–adrenal axis and follows a circadian pattern, producing predictable daily fluctuations in glucocorticoid levels (23, 24). Despite this established framework, how gonadal and adrenal steroid hormones fluctuate relative to one another over time within the same mouse has not been systematically defined in male mice.

By combining low-stress serial arterial sampling with LC–MS/MS-based multi-analyte profiling, the present study extends this foundational work by defining the temporal organization of the circulating steroid network in male mice. This approach reveals a clear distinction between coordinated, rhythmic adrenal steroid output and highly stochastic gonadal testosterone secretion.

### Comparison of testosterone fluctuations in mice vs humans

Before the widespread use of mass spectrometry, studies in rodents have shown that testosterone levels in male mice can exhibit extreme variability (10, 11, 12). Our data support this as we show that testosterone often fluctuates by factors of 10–100 within a short period. On the other hand, in humans, day-to-day testosterone fluctuations are more moderate – typically 10–15% in healthy men (25). Another notable distinction is that testosterone fluctuations in mice, as seen in our data and supported by prior research, are irregular and episodic, which we show can change up to 25-fold within a 12-h period (10, 26). High variability is driven by episodic testicular secretion, influenced by genetics, strain differences, social cues (e.g., dominance, female proximity), and rapid metabolism (10, 26). In humans, testosterone fluctuations follow a predictable diurnal pattern, peaking in the morning and declining 25–35% in the evening (24). Together, these findings emphasize that testosterone regulation in mice is fundamentally episodic rather than rhythmic – a critical distinction that should be carefully considered when extrapolating endocrine findings from murine models to human physiology.

### Testosterone-to-estrogen conversion in male mice

Although testosterone levels in our study often increased more than 100-fold within individual mice, circulating estradiol (E2) remained below the assay’s lower limit of quantification (0.5 pg per 200 μL of charcoal-stripped bovine serum, equivalent to 2.5 pg/mL). This finding confirms the low systemic aromatization capacity previously described in male mice and is consistent with the lack of detectable aromatase expression in most peripheral tissues (27, 28). While aromatase expression is present in specific tissues such as the gonads and brain, the absence of measurable circulating E2 in our data indicates that peripheral testosterone-to-estrogen conversion contributes minimally to circulating levels under basal conditions (27, 28). These results therefore reinforce that, in male mice, estrogen production is primarily gonadal and localized, contrasting with the broader peripheral aromatization observed in humans (29).

### Progesterone and adrenal steroid fluctuations in male mice

While progesterone is often overlooked in male rodents, our findings reveal measurable circulating levels that exhibit variability over time. Although we observed some day-to-day changes in progesterone concentrations, these fluctuations were modest compared to testosterone and corticosterone. Progesterone’s role in male physiology, including potential involvement in neurosteroid pathways and as a precursor to other steroids, warrants further investigation, especially given the subtle but significant variation observed (30, 31). The adrenal gland contains very high progesterone concentrations and represents a major potential source, as recently confirmed by Colldén *et al.* (32). The adrenal gland contains very high progesterone concentrations and represents a major potential source, as recently confirmed by Colldén *et al.* (32). Importantly, progesterone levels did not decrease following orchiectomy in that study, suggesting that the testis is unlikely to be a dominant contributor to systemic progesterone under basal conditions, despite synthesizing progesterone as an intermediate in androgen biosynthesis. Furthermore, Colldén *et al.* showed that tissue progesterone persists even after combined orchiectomy and adrenalectomy and that dietary progesterone can accumulate in adipose tissue and the prostate, indicating that circulating and intra-tissue progesterone arises from multiple sources. Consistent with this framework, our correlation analyses showed that progesterone fluctuations were not synchronized with corticosterone and DOC, suggesting that progesterone regulation is at least partly independent of adrenal steroid rhythmicity. Collectively, these data support a model in which basal circulating progesterone in male mice reflects contributions from adrenal production, peripheral tissue storage and metabolism, and dietary uptake, with relatively stable concentrations that do not mirror the dynamic oscillations of other adrenal steroids.

Corticosterone exhibited a clear diurnal rhythm, with evening concentrations consistently higher than morning values across all sampling days. DOC showed similar directional AM–PM trends, although statistically significant differences were observed only on day 5. The less consistent diurnal rhythmicity of DOC compared with corticosterone may reflect its more rapid metabolic turnover and tighter coupling to acute steroidogenic flux, whereas corticosterone, as the terminal glucocorticoid in mice, likely integrates upstream fluctuations into a more stable circadian profile. The strong positive correlations among these steroids confirm a tightly coupled adrenal steroidogenic network that operates independently from the highly variable testicular output of testosterone. This coordinated adrenal rhythm likely reflects ACTH-driven circadian control, whereas the irregular testosterone fluctuations arise from episodic luteinizing hormone stimulation and rapid metabolic turnover (11).

Our findings are consistent with those of Lucas and Eleftheriou (26), who reported that testosterone exhibits circadian variation in BALB/cBy mice but not in the C57BL/6By strain, further supporting that testosterone secretion in the C57BL/6 genetic background is episodic rather than rhythmic. Together, these findings delineate two distinct modes of endocrine regulation in male mice: a predictable adrenal rhythmicity (23, 24) governing adrenal steroids and a stochastic gonadal secretion pattern driving testosterone variability. This organizational difference underscores the importance of considering both pathway-specific control and sampling time when interpreting murine endocrine data.

Although the present study focused on circulating hormone profiles rather than downstream molecular or physiological responses, these results provide the necessary temporal framework for future work linking hormone dynamics to androgen-responsive gene expression, tissue physiology, and stress or metabolic endpoints. Establishing these connections will be critical for understanding how episodic versus rhythmic steroid signals influence target tissue function in male mice.

### Implications for experimental design and endocrine research

The contrasting dynamics of adrenal and gonadal steroid regulation have direct implications for experimental design and data interpretation in murine endocrine studies. The highly variable and episodic nature of testosterone means that single-point measurements may not accurately represent a mouse’s hormonal state, and assuming extreme values to be outliers could obscure genuine biological variability. In contrast, adrenal steroids exhibit consistent rhythmicity, emphasizing the need to control for the sampling time and light–dark cycle when assessing corticosterone or DOC.

Across the five-day sampling period, mean circulating testosterone concentrations averaged approximately 2 ng/mL, which may serve as a reference point for replacement studies in gonadectomized male mice. However, our findings suggest that restoring testosterone to this mean concentration may not fully replicate physiological conditions, as male mice are conditioned to transient hormonal surges rather than a stable baseline level. The physiological threshold required to elicit specific androgenic effects in mice remains undefined, and determining this relationship should be a priority for future research. These considerations underscore the importance of frequent or time-controlled hormone sampling in experimental design to ensure accurate interpretation of treatment effects and to capture the natural temporal complexity of endocrine regulation in male mice.

An additional consideration is whether tissue steroid measurements may provide a more stable estimate of androgen exposure than circulating testosterone, given the significant fluctuations observed in serum. Tissue hormone levels may reflect integrated uptake, intracellular binding, and local metabolism, potentially buffering rapid pulsatile changes in circulation. Colldén *et al.* demonstrated that correlations between circulating and tissue sex steroid levels vary substantially by tissue, with strong serum–brain and serum–muscle correlations but more modest associations in white adipose tissue and other tissues (16). These findings indicate that local enzymatic activity, tissue-specific metabolism, and storage capacity contribute meaningfully to intra-tissue steroid levels. Thus, while tissue measurements may provide complementary information regarding androgen exposure, they are regulated by both systemic supply and local steroidogenic dynamics. Direct longitudinal comparisons of circulating and tissue testosterone under basal conditions would be required to determine whether tissue measurements provide a more stable index of androgen status in male mice.

### Limitations

This study was performed in unstressed adult male C57BL/6J mice housed under a 12:12 light–dark cycle. Therefore, the hormone patterns described here may not apply to other strains, females, different age groups, alternative light schedules, or stress conditions. Sex, genetic background, and environmental factors are known to influence steroid hormone regulation and could produce different temporal profiles. In addition, diurnal sampling was conducted at two time points, capturing the early light phase and the period immediately preceding lights off; although these data demonstrate robust diurnal variation consistent with established circadian regulation, additional sampling during the dark phase would be required to fully characterize the 24-h hormonal pattern. Mice were singly housed after catheter implantation to protect the vascular access system and prevent male–male aggression. Although this was necessary for technical reasons, single housing is not natural for mice and may influence endocrine function, particularly testosterone levels. All animals were maintained under the same conditions to minimize variability, but future studies examining different social and environmental contexts would help further define these hormone dynamics.

## Conclusion

This study provides a comprehensive temporal characterization of steroid hormone dynamics in adult male mice using longitudinal, within-animal sampling and high-sensitivity LC–MS/MS analysis. Our findings reveal two distinct modes of endocrine regulation: a coordinated adrenal rhythmicity governing corticosterone and DOC and a pulsatile gonadal pattern driving testosterone variability. Progesterone remained comparatively stable and largely independent of these pathways. These data establish a physiological framework for interpreting endocrine outcomes in male mice and underscore the importance of considering both temporal variability and pathway-specific regulation when designing and analyzing experimental studies. Future work integrating these hormone profiles with downstream gene expression and tissue-level responses will be critical to understanding how episodic versus rhythmic steroid signaling shapes male physiology.

## Supplementary materials



## Declaration of interest

The authors declare that there is no conflict of interest that could be perceived as prejudicing the impartiality of the work reported.

## Funding

This work was supported by the NIH grant to RTE (Grant No. K01-AT010348) and the NSF-MRI program under Award No. 1828059.

## Ethical statement

All animal experiments performed in this study were approved by Vanderbilt University’s IACUC.
