# Supplementary material for: Temporal organization of gonadal and adrenal steroid fluctuations in male mice
Source: Endocr Connect. 2026 Mar 23;15(3):e260091. doi: 10.1530/EC-26-0091 (PMC13034558; doi:10.1530/EC-26-0091)
Supplement: Supplementary file 1 [file supplementary_materials.pdf]

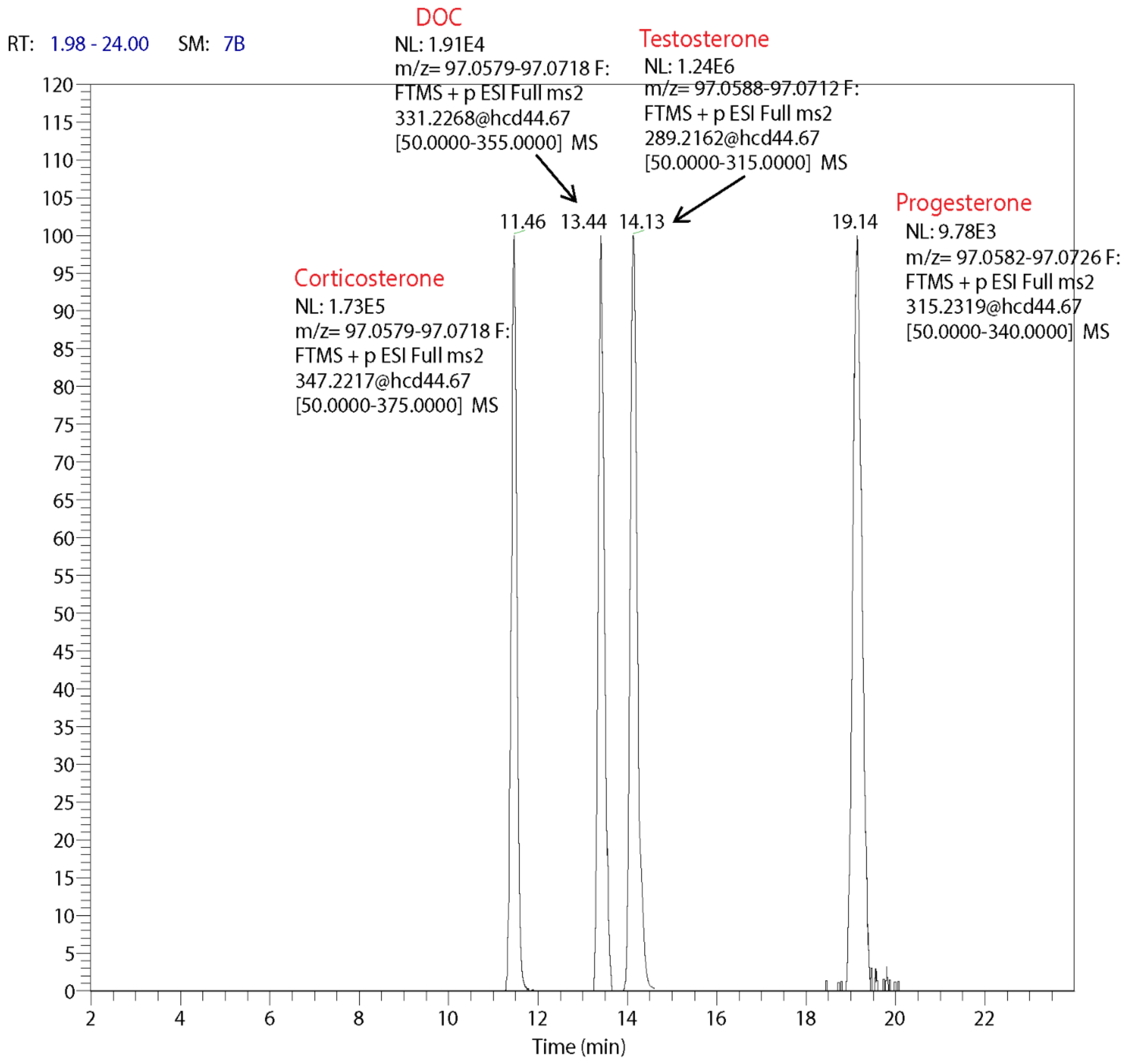

Supplementary Figure 1. Representative extracted ion chromatograms for testosterone, progesterone, corticosterone, and deoxycorticosterone (DOC) from mouse plasma samples analyzed by LC–MS/MS are shown. Chromatograms are displayed with signal intensities normalized to 100%.

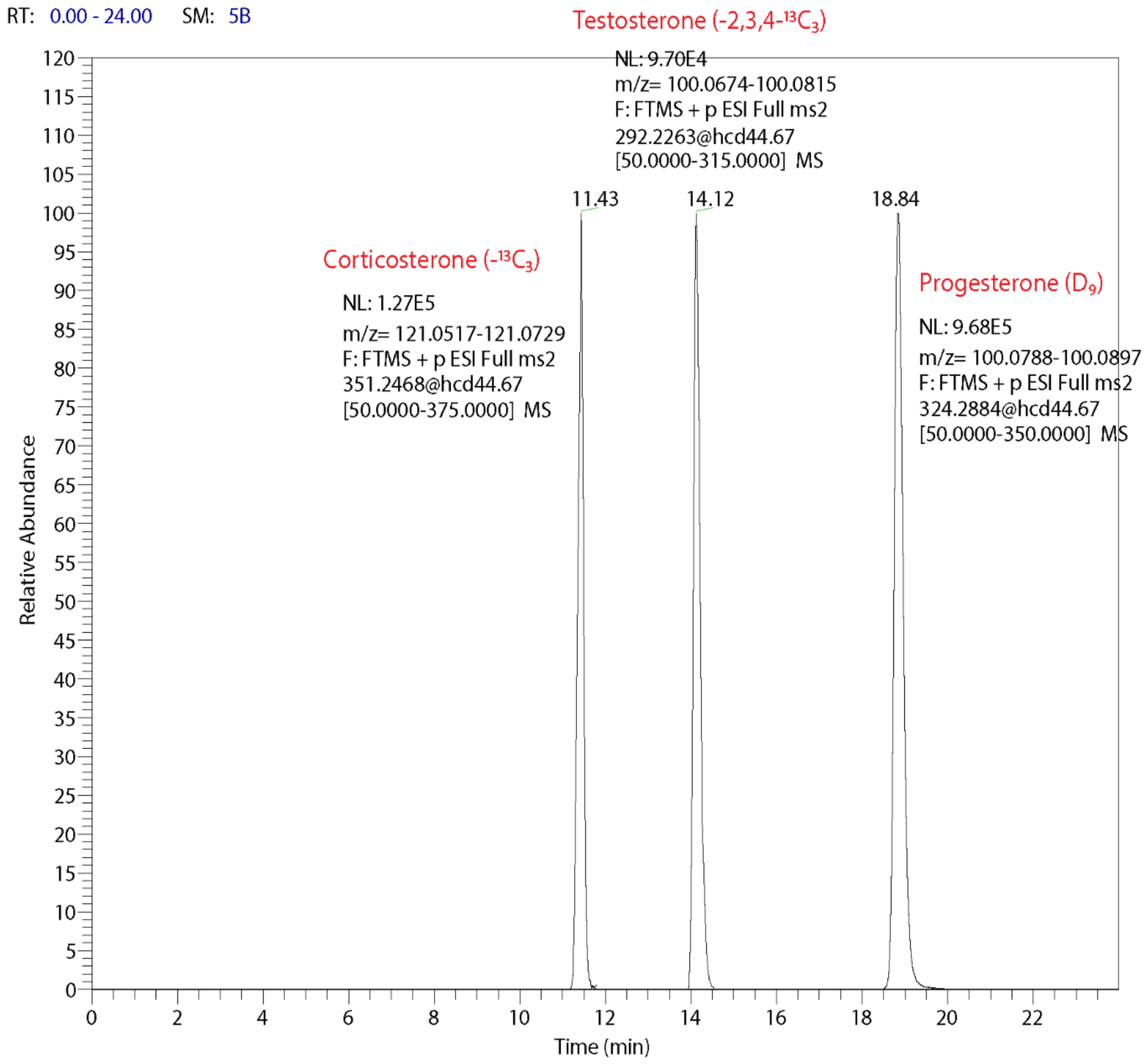

Supplementary Figure 2. Representative extracted ion chromatograms for the stable isotope–labeled internal standards corresponding to testosterone (-2,3,4-$^{13}C_3$), progesterone (D_9), and corticosterone (-$^{13}C_3$) are shown from mouse plasma samples analyzed by LC–MS/MS. The corticosterone internal standard was used for quantification of both corticosterone and deoxycorticosterone (DOC). Signal intensities are normalized to 100%.
